# Digital health technology combining wearable gait sensors and machine learning improve the accuracy in prediction of frailty

**DOI:** 10.3389/fpubh.2023.1169083

**Published:** 2023-07-20

**Authors:** Shaoyi Fan, Jieshun Ye, Qing Xu, Runxin Peng, Bin Hu, Zhong Pei, Zhimin Yang, Fuping Xu

**Affiliations:** ^1^The Second Clinical College of Guangzhou University of Chinese Medicine, Guangzhou, China; ^2^School of Civil Engineering and Transportation, South China University of Technology, Guangzhou, China; ^3^Division of Translational Neuroscience, Department of Clinical Neurosciences, Hotchkiss Brain Institute, Alberta Children’s Hospital Research Institute, Cumming School of Medicine, University of Calgary, Calgary, AB, Canada; ^4^Department of Neurology, First Affiliated Hospital of Sun Yat-Sen University, Guangzhou, China; ^5^The Second Affiliated Hospital of Guangzhou University of Chinese Medicine, Guangdong Provincial Hospital of Chinese Medicine, Guangzhou, China

**Keywords:** digital health technology, wearable sensor, machine learning, prediction model, frailty, gait

## Abstract

**Background:**

Frailty is a dynamic and complex geriatric condition characterized by multi-domain declines in physiological, gait and cognitive function. This study examined whether digital health technology can facilitate frailty identification and improve the efficiency of diagnosis by optimizing analytical and machine learning approaches using select factors from comprehensive geriatric assessment and gait characteristics.

**Methods:**

As part of an ongoing study on observational study of Aging, we prospectively recruited 214 individuals living independently in the community of Southern China. Clinical information and fragility were assessed using comprehensive geriatric assessment (CGA). Digital tool box consisted of wearable sensor-enabled 6-min walk test (6MWT) and five machine learning algorithms allowing feature selections and frailty classifications.

**Results:**

It was found that a model combining CGA and gait parameters was successful in predicting frailty. The combination of these features in a machine learning model performed better than using either CGA or gait parameters alone, with an area under the curve of 0.93. The performance of the machine learning models improved by 4.3–11.4% after further feature selection using a smaller subset of 16 variables. SHapley Additive exPlanation (SHAP) dependence plot analysis revealed that the most important features for predicting frailty were large-step walking speed, average step size, age, total step walking distance, and Mini Mental State Examination score.

**Conclusion:**

This study provides evidence that digital health technology can be used for predicting frailty and identifying the key gait parameters in targeted health assessments.

## Introduction

As the population ages, frailty is becoming a major challenge for public healthcare. Frailty is a condition that affects many older people and is characterized by a decline in physical function, decreased resilience to stressors, and a higher risk of negative health outcomes such as falls, hospitalization, and death (1). Patients with frailty commonly show multifaceted clinical symptoms, phenotypic heterogeneity, and fluctuating manifestations that challenge the comprehensive appraisal of the condition (2, 3). Undiagnosed frailty is common in older people, since it typically has no explicit connection to a defined medical issue; therefore, frailty frequently remains untreated until later stages. Early detection of the risk of frailty is essential especially in the early stages, as such identification would facilitate the implementation of treatments to slow down declines and reduce adverse outcomes.

Currently, the prediction of frailty is performed using questionnaires and tests of physical activity, muscle strength or gait. The most widely used assessment is the fried performance (FP) test (4), which includes measures of weight loss, exhaustion, low physical activity, weakness, and slow walking speed. However, the FP test only evaluates the physical aspects of frailty, while frailty is acknowledged to be a multifaceted state that includes not only physical dimensions, but also social, cognitive, and psychological dimensions (5, 6). Gait-related mobility is a key physical ability that has been linked to frailty and is often used as a predictor of future health outcomes (7, 8).

During the aging process, the loss of function is intrinsic to all the physiological systems, including the Central and peripheral nervous systems, musculo-skeletal system, and cardiopulmonary system. The most dramatic and significant changes is the decline in limb muscle, which can lead to changes in gait parameters such as walking speed, cadence, and stride-length (9, 10). Gait speed, which is a quantifiable index of ambulatory ability and a major predictor of future health outcomes (11), is commonly used as an outcome in the research of frailty.

Advances in technologies associated with wearable devices has enabled the collection of more precise parameters regarding other spatial and temporal gait variables in addition to the commonly observed gait speed. These technologies are valid and practical, and they provide a promising, cost-effective digital method in standardizing the data collection and analysis of gait function with improved efficiency and accessibility both in the clinical setting and within seniors’ living communities (12–14).

With improved multidisciplinary diagnostic approaches, some researchers have predicted frailty status based on CGA, but this approach has several limitations. For example, questions within the CGA frequently require advanced knowledge and thus might not accurately reflect the mental status of subjects. Concurrently, with the evolution of computer science and artificial intelligence, many researchers would like to predict frailty using ML approaches (Table 1) (15–23). Previous studies in this regard have commonly utilized easy-to-access epidemiological datasets or electronic health records to construct ML algorithms (24); the nature of these databases mean that these algorithms are potentially limited to the use of a single dimension. Furthermore, little research has analyzed Asian populations, which have very different socio-economic profiles as compared with Western populations (16).

**Table 1:** Previous researches regarding frailty risk prediction by machine learning.

Reference	Number of variables	Machine learning model	Data type	Outcome
Ambagtsheer et al. (15)	70	SVM, DT, and KNN	Administrative records	SVM (sensitivity of 97.8%, specificity of 89.1%), DT (sensitivity of 63.0%, specificity of 21.4%), and KNN (sensitivity of 63.0%, specificity of 71.7%)
Aponte-Hao et al. (16)	75	ENLR, SVM, KNN, NB, DT, RF, XGBoost, and ANN	Administrative records	In terms of AUROC, ENLR (0.82), SVM (0.80), KNN (0.66), NB (0.74); DT (0.77), RF (0.81), XGBoost (0.83), and ANN (0.78)
Le Pogam et al. (17)	18	LR, RF, and SVM	Electronic medical records	In terms of AUROC, best-subsets LR (0.71), RF (0.66), SVM (0.58)
Tarekegn et al. (18)	58	ANN, GP, SVM, RF, LR, and DT	Administrative records	ANN classifier generated the optimal prediction results for mortality: Accuracy within ANN (0.78),SVM (0.79), RF (0.78), LR (0.78), DT (0.75)
Koo et al. (19)	27	SVM, RF, and GB	Electronic medical records	SVM (Precision of 88.9%), RF (Precision of 92.3%), and GB (Precision of 88.0%)
Williamson et al. (20)	3,761	LR	Electronic medical records	Sensitivity of 36.1%, specificity of 62.9%, PPV of 17.3%, and NPV of 82.1%
Park et al. (21)	16	LR	Pendant Sensor data	AUROC of 0.80, sensitivity of 72.2%, specificity of 70.0%, and accuracy of 71.3%
Kraus et al. (22)	9	RF, KNN, RF	Insole Sensor data	In terms of AUROC, RF (0.92), KNN (0.80)
Minici et al. (23)	25	RF, NB, LR, SVC, MLPC	Wrist Sensor data	In terms of AUROC, RF (0.80), NB (0.87), LR (0.73), SVC (0.64), MLPC (0.71)

SVM, support vector machine; DT, decision tree; KNN, K-nearest neighbors; ENLR, elastic net logistic regression; NB, naive bayes; XGBoost, extreme gradient boosting; LR, logistic regression; BS-LR, best-subsets logistic regression; RF, random forest; ANN, artificial neural network; MLPC, multilayer perceptron classifier; KFACS, Korean Frailty and Aging Cohort Study.

Therefore, the objectives of this study were to (A) obtain the relevant gait parameters using wearable sensor and to analyze their association with deterioration of physical function and evolution into frailty, and to (B) develop an ML frailty risk prediction model suited for an Asian population, using a combination of both wearable sensor-based gait analysis and CGA.

## Materials and methods

### Study design, participants, and features

In total, we consecutively recruited 214 community-dwelling volunteers from the course of the anti-aging study, a prospective cohort study conducted to investigates the association of frailty with health. All subjects were recruited through advertisements in two ways. First, recruitment was conducted at four communities centers in Guangzhou (the capital of the Guangdong Province in the southeastern region of China). Second, with the assistance of the District Health Center and GPs clinics, recruitment invitations was handed out via online announcement to older individuals on Wechat platforms. Eligibility criteria for participants were: aged 60–95 years, having adequate auditory and visual acuity, and the ability to ambulate with or without any walking aids or assistance of others. Exclusion criteria included orthopedic or neurological complications or other relevant medical conditions that might restrict walking speed and natural movement. All subjects were required to complete CGA including but not limited to a standardized questionnaire that collected demographic information, medical and medication records, as well as multidimensional clinical assessment, including anthropometric evaluation, emotional evaluation, and neuropsychological evaluation.

The parameters of CGA included the patients’ demographic data, including age, gender, education level, marital status, employment position and measurement data. The clinical measurement data involved multimorbidity (defined by the coexistence of >2 chronic conditions), polypharmacy (defined as currently using >5 drugs), depression disorder (25) (defined by scores ≥10 on the 9-item Patient Health Questionnaire, PHQ-9), anxiety disorder (26) (defined by scores ≥10 on the 7-item Generalized Anxiety Disorder, GAD-7), cognitive function (27, 28) (assessed by the Mini-Mental State Examination, MMSE and the Chinese versions of the Montreal Cognitive Assessment, MoCA-BC) and neuropsychiatric function (29, 30) (assessed by the Mild Behavioral Impairment Checklist, [MBI-C]). A detailed description of how these features were defined is also provided in Table 2.

**Table 2:** Features of cohort characteristics used for machine learning.

Demographics	Data type	Clinical measures	Data type	Walking test	Data type
Age	Numeric	Exhaustion	Binary	Total step walking distance	Numeric
Education level	Categorical	Sleep quality	Binary	Average walking speed	
Gender	Binary	Anxiety	Binary	Total cadence	
Marital status/partnerships	Categorical	Depression	Binary	Large step distance	
Employment position	Categorical	Cognition test	Numeric	Large step walking time	
Medical history		Energy expenditure	Binary	Large step walking speed	
Medical record	Binary	Anthropometry		Large step cadence	
Medications/supplements	Binary	Grip strength	Numeric	Average step size	
Unintentional weight loss	Binary	Body mass index (BMI)	Numeric	Average step time	

### Walking test

To obtain an objective and quantitative assessment of gait parameters, all participants were instructed to complete a 6-min walk test (6MWT) using a wearable sensor (Ambulosono Sensor System) (13, 31). The sensor was connected to the iOS Gait Reminder App, which can issue auditory instructions while continuously recording step size via an iOS gyroscope and accelerometers, after corrections for limb length, angular excursion, signal filtering, and drift. Participant were instructed to walk independently if possible, and were permitted to use walking aid (e.g., walker or cane) if needed.

### Frailty assessment

The outcome measures used to categorize no-frailty and frailty was assessed utilizing the five components specified in the FP test (4), including: self-reported unintentional weight loss of 10 pounds or more within the last year; self-reported exhaustion; slowness stratified by gender and height; weakness via grip strength test using a hand dynamometer; and low physical activity based on the short version Minnesota Leisure Time Activity questionnaire (32). FP scores range from 0 to 5 points, with higher scores indicating more severe frailty. Based on the results, individuals with scores of 0 through 2 were categorized into the no-frailty group, and those with scores of 3 or more were included in the frailty group. This scale has exhibited high validity and has become a gold standard for classifying frailty in older adults (33).

### Machine learning models

In this study, five widely accepted and extensively used supervised ML models were applied: random forests (RF); decision trees (DT); naïve Bayes; neural network (NN) and stochastic gradient descent (SGD).

DT is a powerful ML algorithm capable of performing classification tasks. Advantages of DT include simplicity, interpretability, ability to model nonlinear data and ability to handle outliers during training (34), but weaknesses of the simple decision tree are instability and a risk of over fitting. RF, a popular ensemble classification method, combines multiple learning algorithms to achieve better performance. RF models generally outperform those generated by DT in terms of accuracy. Naive Bayes is a supervised probabilistic machine learning algorithm for probabilistic classification that relies on Bayes’ theorem with an assumption of strong independence between the input features (35). Neural network is a mathematical computing model that imitates the construction of biological neural networks and that is commonly used in classification tasks with various applications (36).We also attempted to apply SGD (37), an algorithm for optimization problems arising in high-dimensional inference tasks.

### Experimental setting

**Figure 1:**
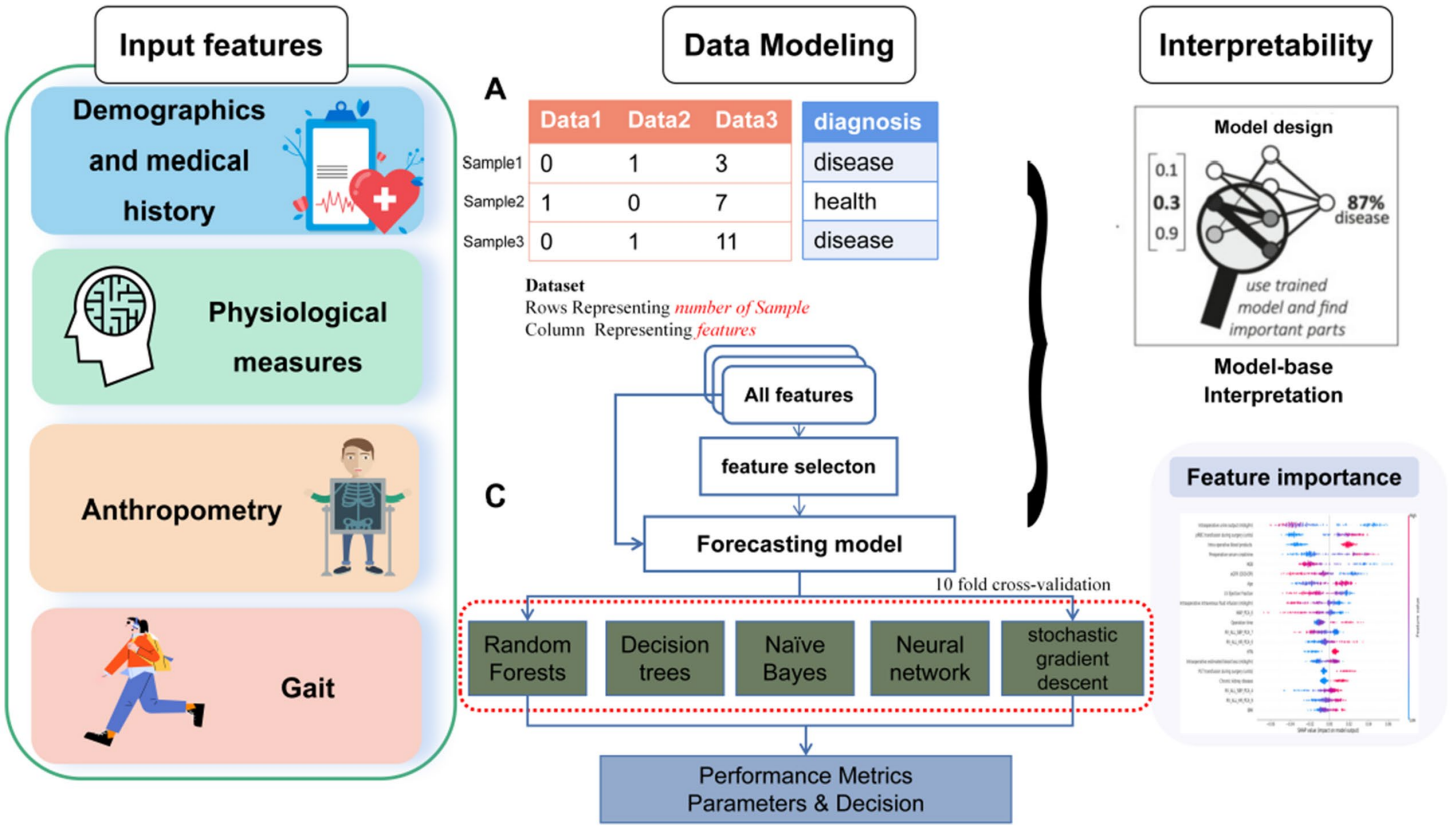
Overall data acquisition, feature extraction, feature selection, data classification analysis and machine learning flow for the classification of frailty.

An overview of the ML approach used in this study is shown in Figure 1. After imputation of missing values by multiple interpolation, the values of input features were standardized to ensure that each feature had the same influence on the cost function in designing the ML models. In this study, the data were randomly divided, with 70% used for training and the remaining 30% used as test data. Within the 70% training set, the data was split into ten random folds for cross validation to guard against overfitting. The rigorous use of holdout method with random resampling and stratified k-fold cross-validation ensured the validity and generalizability of the findings, and helped to mitigate potential biases in the analysis. We used average values as they are robust to outlying predictions.

### Feature selection

When not all features have significant class discrimination information, using feature selection methods can help to remove the irrelevant and redundant features. This method can reduce the computational time and improve classification performance. To determine the lowest number of demographic features, clinical features and sensor-derived gait sequence features required to best identify frailty, optimal feature selection using either the independent samples t-test or the chi-square test was applied, depending on each feature’s types. Sixteen of the features showed a significant difference between the no-frailty and frailty groups, and thus they were used as independent variables for optimal feature selection.

### Performance measures

The performance of the ML models was evaluated using AUC score, sensitivity, specificity, precision, F1-score and accuracy. We aimed to find the simplest model that achieves the highest accuracy. As the original training data were imbalanced (13.1% frailty), such data can result in biased estimates of training performance; hence, with the best performing model chosen by average AUC score, which can be understood as the probability that a randomly chosen no-frailty patient will have a score lower than a randomly chosen frailty patient. SHapley Additive exPlanation (SHAP) values were used to provide consistent and locally accurate attribution values for each feature within each prediction model (38).

### Statistical analyses

All statistical analyses and calculations were performed using R software and Python (version 3.9.7; Python Software Foundation). The categorical variables were expressed as total numbers and percentages, with the chi-square test used for comparison between groups. Normally continuous variables were expressed as x̅ ± s, and the independent samples *t*-test for was used for comparisons between groups; non-normally continuous variables were expressed as median and IQR, with a Mann–Whitney U test used for comparisons between groups. The inspection level α = 0.05, and we considered any difference statistically significant if *p* < 0.05.

## Results

### Demographic and clinical characteristics

Table 3 compares the detailed demographic and clinical characteristics of each group. A total of 214 subjects were included in the study, among which 28 (13.1%) subjects were classified into the frailty group. Compared with the no-frailty group, subjects in the frailty group were of older age (74.3 ± 8.4 vs. 68.1 ± 6.1 years) and had lower BMI (22.2 ± 2.7 vs. 23.5 ± 2.9). Moreover, the frailty group had a higher proportion of comorbid conditions (74.4% vs. 31.7%), polypharmacy (35.7% vs. 8.6%) and depression (32.1% vs. 14.0%) with significant difference. The results also showed that the scores on the Mini Mental State Examination (MMSE) and the Montreal Cognitive Assessment from subjects in the frailty group were significantly lower than those of subjects in the no-frailty group (*p* < 0.01). There were no significant differences in gender or education between two groups (Table 3).

**Table 3:** Demographics and clinical characteristics of participants stratified by frailty status.

Variable	Frailty status			
	All (*n* = 214)	No-frailty (*n* = 186)	Frailty (*n* = 28)	*p*-value
Age, mean (SD)	68.9 (6.7)	68.1 (6.1)	74.3 (8.4)	<0.001
Female	156 (72.9)	139 (74.7)	17 (60.7)	0.120
Education				0.212
Primary or lower	119 (55.6)	104 (55.9)	15 (53.6)	
Completed high school	69 (32.2)	57 (30.6)	12 (42.9)	
At least some college	26 (12.1)	25 (13.4)	1 (3.6)	
Marital status				0.782
Married	173 (80.8)	152 (81.7)	21 (75.0)	
Divorced	32 (15.0)	24 (14.5)	5 (17.9)	
Widowed	9 (4.2)	7 (3.8)	2 (7.1)	
Comorbid conditions >2	79 (36.9)	59 (31.7)	20 (71.4)	<0.001
Polypharmacy	26 (12.1)	16 (8.6)	10 (35.7)	<0.001
BMI, mean (SD)	23.3 (2.9)	23.5 (2.9)	22.2 (2.7)	0.026
Insomnia(PSQI>7)	121 (56.5)	104 (55.9)	17 (60.7)	0.633
Depression (PHQ-9 ≥ 10)	9 (8.4)	26 (14.0)	9 (32.1)	0.015
Anxiety (GAD-7 ≥ 10)	15 (7.0)	13 (7.0)	2 (7.1)	0.976
MBI-C, mean (SD)	5.1 (3.8)	5.0 (3.8)	5.9 (3.6)	0.208
MMSE, mean (SD)	26.2 (3.0)	26.6 (2.8)	23.7 (3.5)	<0.001
MoCA-B, mean (SD)	24.2 (3.4)	24.5 (3.2)	22.2 (3.6)	0.001

SD, standard deviation; BMI, body mass index; PSQI, Pittsburgh Sleep Quality Index; PHQ-9, Patient Health Questionnaie-9; GAD-7, General Anxiety Disorder Screener-7; MBI-C, Mild Behavioral Impairment Checklist; MMSE, Mini-mental State Examination; MoCA-B, Chinese versions of the Montreal Cognitive Assessment; Results presented as *n* (%) unless otherwise noted. Chi-square tests were used for categorical variables, whereas *t*-tests were used for continuous variables.

### Characteristics of the gait features in each study group

Table 4 shows gait parameters in relation to the presence of frailty. In comparison to subjects in the no-frailty group, those in the frailty group had slower gait speed (57.3 ± 17.6 vs. 70.1 ± 15.2 m/min), lower step walking distance (338.6 ± 96.7 vs. 420.5 ± 91.3 m), lower total cadence (113.5 ± 11.7 vs. 120.4 ± 15.2 step/min), shorter step size (0.508 ± 0.143 vs. 0.593 ± 0.107 m), and higher average step time (0.533 ± 0.059 vs. 0.502 ± 0.051 s).

**Table 4:** Characteristics of the gait sequence features in each study group.

Variable	Unit	Frailty index status			
		All (*n* = 214)	No-frailty (*n* = 186)	Frailty (*n* = 28)	*p*-value
Total step walking distance (m)	m	409.8 (95.9)	420.5 (91.3)	338.6 (96.7)	<0.001
Large step distance (m)	m	402.5 (103.4)	413.7 (99.0)	328.6 (103.1)	<0.001
Average gait speed (m/min)	m/min	68.5 (16.2)	70.1 (15.2)	57.3 (17.6)	<0.001
Large step walking speed	m/min	69.0 (15.7)	70.8 (14.6)	57.4 (17.6)	<0.001
Total cadence (step/min)	Step/min	119.5 (14.9)	120.4 (15.2)	113.5 (11.7)	0.023
Large step cadence	Steps/min	117.7 (15.4)	118.9 (15.4)	109.6 (13.1)	0.003
Average step size	m	0.582 (0.115)	0.593 (0.107)	0.508 (0.143)	<0.001
Average step time	s	0.506 (0.053)	0.502 (0.051)	0.533 (0.059)	0.004
Step size variance (Median)	n	0.007 [0.003–0.019]	0.006 [0.003–0.019]	0.010 [0.003–0.023]	0.184^†^
Step time variance (Median)	n	0.012 [0.009–0.021]	0.012 [0.008–0.020]	0.018 [0.011–0.036]	0.002^†^

^†^Tested using the Mann–Whitney U test.

### Performance of machine learning approaches

We performed feature selection analyses among the various feature categories to investigate and identify crucial feature signatures for our models. The input data used were the gait features based on the outputs from wearable sensor and the demographic and clinical features derived from CGA. As illustrated in Table 5, the ML models achieved up to 63.5% accuracy, 88.2% specificity, 56.5% sensitivity, 98.4% precision and an F1-score of 65.0% using demographic and clinical features. When using the gait features, the ML models achieved up to 65.6% accuracy, 83.8% specificity, 65.3% sensitivity, 97.8% precision and F1-score of 65.6%. In comparison, the ML models that employed all features achieved the highest performance, with up to 71.1% accuracy, 89.5% specificity, 61.3% sensitivity, 97.0% precision, and an F1-score of 68.0%.

**Table 5:** Performance summary of ML models for frailty classification using different features set.

Models	Accuracy	Specificity	Sensitivity	Precision	F1-score
**Demographic and clinical features**					
Random forests	60.56	88.21	54.42	98.35	65.01
Decision trees	63.50	71.37	56.53	90.17	64.30
Naive bayes	56.63	61.81	54.25	67.62	53.22
Neural network	52.53	65.23	51.75	84.59	53.82
Stochastic gradient descent	58.75	82.13	55.63	86.42	58.16
**Gait sequence features**					
Random forests	63.22	83.80	54.81	97.84	65.57
Decision trees	63.60	70.81	56.27	91.17	64.57
Naive bayes	60.47	60.06	55.14	76.23	59.64
Neural network	47.49	56.37	47.12	74.54	44.96
Stochastic gradient descent	53.75	78.35	52.77	91.00	58.62
**All features**					
Random forests	69.58	89.46	58.54	96.95	68.80
Decision trees	71.11	78.28	60.86	92.35	68.68
Naive bayes	67.98	66.74	61.30	75.38	63.06
Neural network	67.90	75.60	58.73	89.75	66.80
Stochastic gradient descent	58.42	78.22	53.30	92.54	62.69
**Selected features**					
Random forests	66.58	95.69	57.38	98.76	67.74
Decision trees	68.74	80.14	59.45	93.10	67.88
Naive bayes	65.35	73.88	57.31	86.33	64.29
Neural network	51.46	61.74	50.51	77.42	48.27
Stochastic gradient descent	59.00	78.71	55.70	89.50	61.43

**Figure 2:**
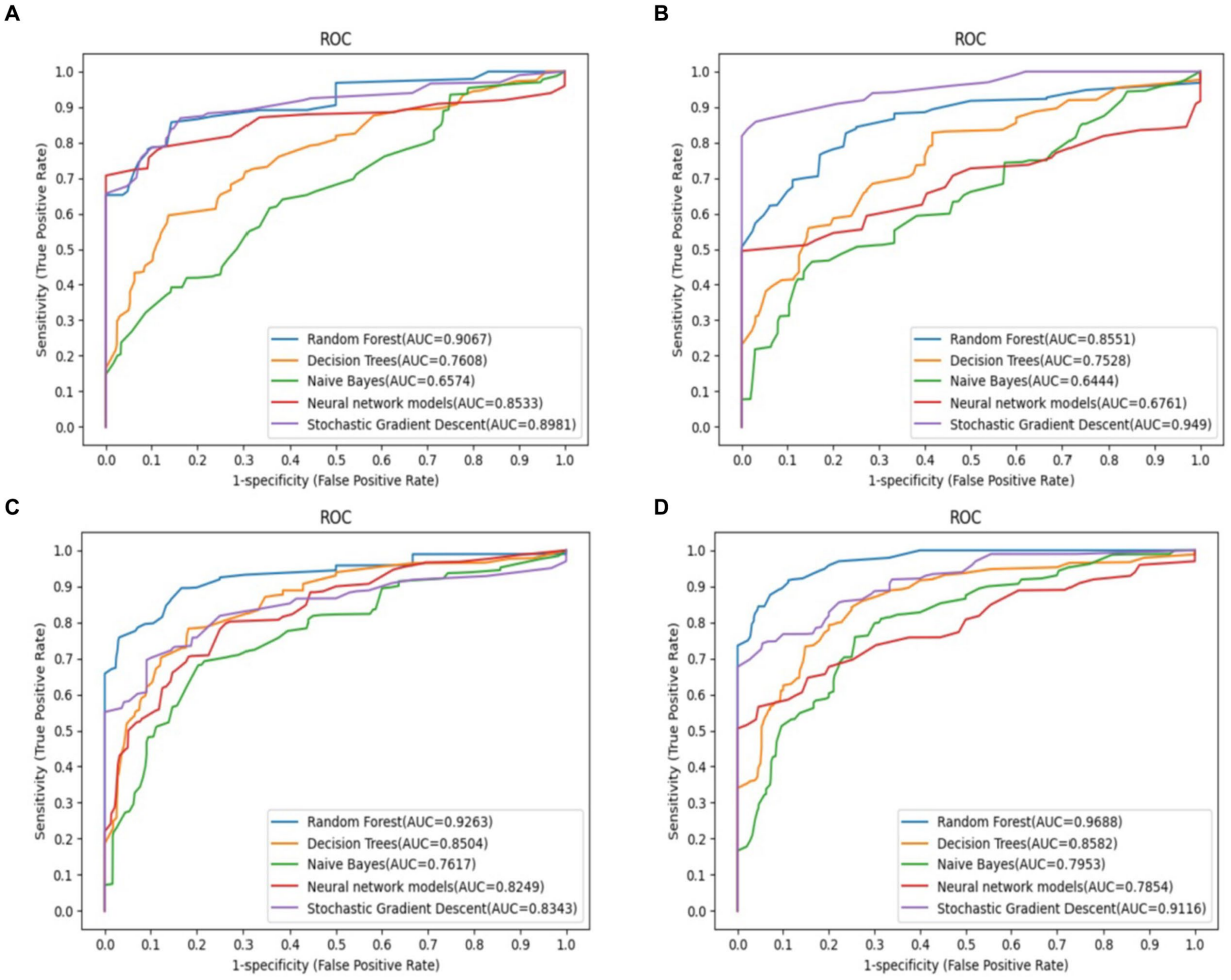
Predictive performance of different feature selection in predict the outcomes of frailty model. The RF model demonstrated the most favorable performance. **(A)** Demographic and clinical prediction (AUC = 0.907), **(B)** gait sequence prediction (AUC = 0.855), **(C)** all feature prediction (AUC = 0.926), **(D)** selected feature prediction (AUC = 0.969).

The receiver operating characteristic (ROC) curves of each ML model using different features are shown in Figure 2. All ML models achieved significant improvements in discrimination by using selected features as compared to the indiscriminate use of all features. In particular, RF exhibited an AUC gain of 4.3% when using selected features as compared to using all features, a gain of 6.2% when using selected features as compared to all demographic and clinical features, and a gain of 11.4% when using selected features as compared to using all gait sequence features. Since the RF model outperformed other ML models according to accuracy and AUC, it was selected for all downstream analyses.

### Feature importance

**Figure 3:**
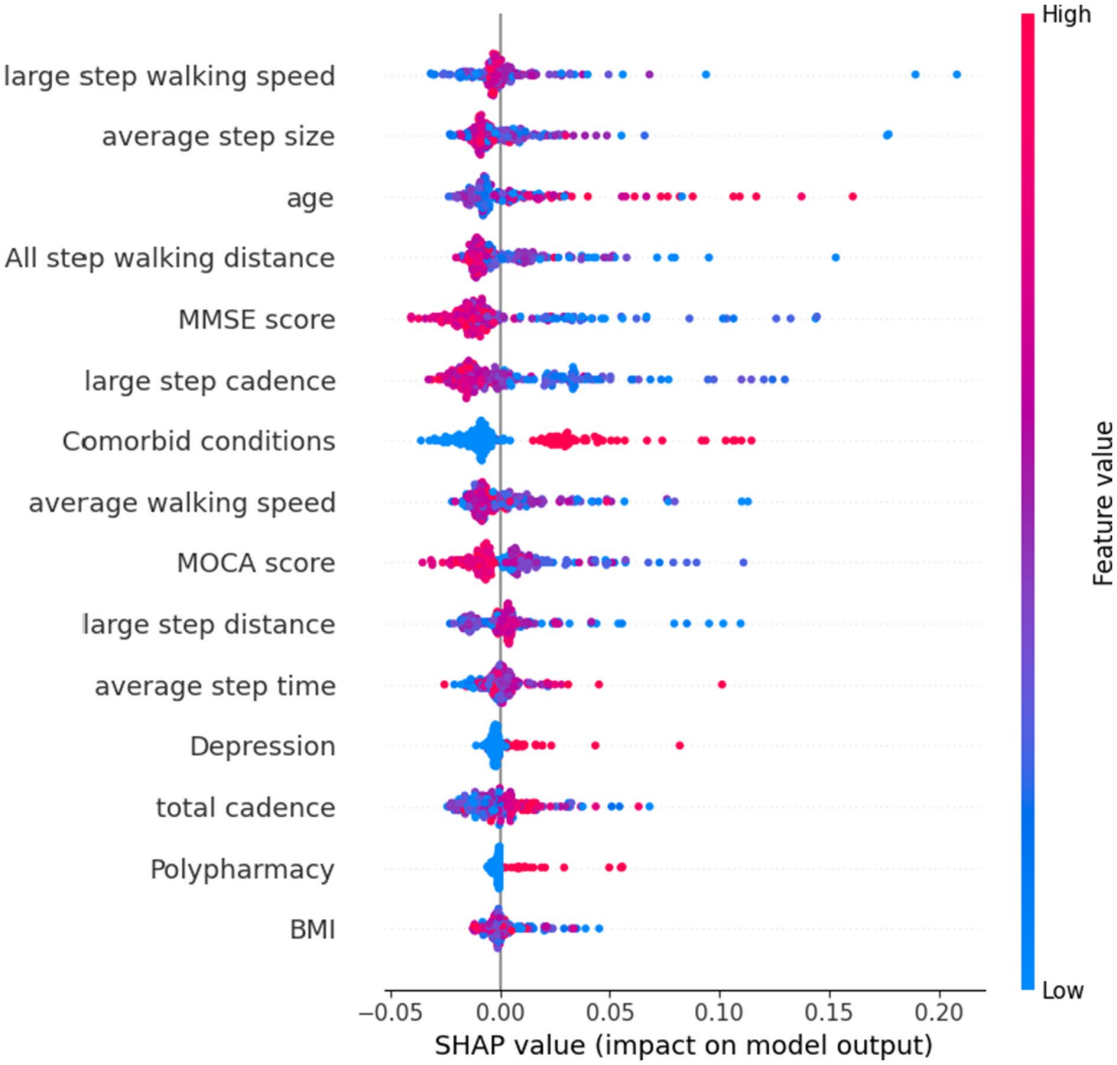
SHAP plot of top features influencing our model’s prediction of frailty using all features.

Figure 3 shows the SHAP summary plot of RF using selected features, with the features contributing to the model in descending order of average absolute SHAP values. This plot depicts the relationships of values to SHAP values in the training dataset. According to the prediction model, the higher the SHAP value of a feature, the more likely frailty becomes. As observed in the plot, the five most important predictors in the prediction model were large step walking speed, average step size, age, total step walking distance, and MMSE score.

## Discussion

Frailty is a common condition among older adults that is characterized by a decline in physical and cognitive function and an increased risk of adverse health outcomes. Identifying frailty on the early stage can help healthcare professionals implement hierarchical strategies to prevent or delay its onset and manage potential conditions. In this study, we adopted a set of analytical and machine-learning approaches to analyze the relationship between frailty and a combination of CGA and wearable sensor-derived gait parameters in community-dwelling older adults.

To achieve this, we used statistical methodologies to extract a subset of uncorrelated components associated with frailty predictors, which were then used to independently identify and visualize multiple dimensions associated with frailty. Our results indicate that machine learning methods are effective in predicting frailty and that using a combination of both CGA characteristics and wearable sensor data improved the performance of our model compared to using either features separately. This multifaceted combination of features provides a comprehensive perspective on frailty, allowing the model to capture the intricate relationship between various factors.

Our final model, which was processed using a random forest machine learning method, achieved an impressive area under the curve (AUC) score of 0.926, indicating a high level of accuracy compared with previous estimates, ranging from 0.58 up to 0.92 (15–23). In contrast to other traditional predictive formulas, such as the electronic medical records-based model, administrative records-based model, and sensor-based model, the classification results of our model outperformed other traditional models. This enhanced performance may be attributed to our study design and to the synergistic approach that combined CGA and wearable sensor data, accompanied by rigorous feature selection and optimization processes.

Furthermore, we also calculated the accuracy of each model when processing different complements of patient characteristics, in addition to overall model performance. We found that using a narrower set of carefully selected features achieved significant improvements in discrimination relative to models that included all available features. This reduced the dimensionality of our dataset and allowed our model to focus on the most relevant features for predicting frailty. In previous study, Williamson and colleagues (20) reported that frailty was predicted using a large number of features of electronic medical record database without feature selection as effectively, which can lead to overfitting and reduced generalization of the model.

This limitation of the number of features needed would be helpful in clinical settings due to difficulties in collecting consistent data from older patients, especially with functional data or data indicating cognitive and emotional status. Notably, the top five features found to be important in predicting frailty were step walking speed, average step size, age, total step walking distance, and MMSE score. These results are consistent with previous research that has identified gait and physical activity parameters measured by wearable sensors as being associated with physical frailty and has found that certain gait parameters, such as percentage time standing, percentage time walking, walking cadence, and longest walking bout, are effective digital biomarkers for identifying frailty (21, 39). Gait stability, as determined by double-limb support time, step time and stride time, and long short-term memory have also been found to produce the highest discriminative power in identifying frailty using the RF model (40). Accordingly, our findings suggest that healthcare professionals treating older patients should focus on these five features, as they may indicate the risk of future frailty.

In particular, the strength of the gait parameters in our analysis, accounting for 50% of the predictive power, demonstrates the importance of these motor characteristics as a measure of frailty. The 6MWT is a valid, reliable and sensitive measure of functional performance capacity that has been found to be useful in evaluating frailty in older adults (41). Using digital methods to perform the 6MWT, such as wearable sensors or GPS devices, an economical and convenient way to provide diagnostic and clinical information, can significantly improve the accuracy and reliability of the test results compared to manual methods (42, 43).

CGA has also been used to identify risks of adverse events such as cognitive impairment, mortality, functional decline, surgical complications, and chemotherapy toxicity among frailty patients (44, 45). This is the first study ever done to show the benefits of utilizing a combination of CGA and wearable sensor data in predicting frailty. Our research adds to the existing knowledge by revealing the efficacy of employing an integrated approach, using both CGA and wearable sensor data in predicting frailty and a limited number of judiciously selected features in a machine learning model. It also highlights the importance of gait-related measures for frailty prediction. Previous studies have used various artificial intelligence (AI) models to analyze frailty and predict frailty risk in older adults using different types of data, including clinical records (15–18, 24, 46), physical function data (47, 48), and wearable sensor data (21, 22, 49). Additionally, the clinical implications of our method were summarized as follows:

A. The integration of both CGA and wearable sensors objectively measure gait parameters offers healthcare professionals comprehensive understand of frailty risk, enabling early intervention and better management of pre-clinical condition to prevent adverse health outcomes.

B. In terms of feature selection, our study identified the five most important indexs in the prediction model as large step walking speed, average step size, age, total step walking distance, and MMSE score. Clinicians can prioritize these factors when assessing frailty risk in older patients, allowing for a more effective and targeted approach that streamlines data collection and reduces the burden of frailty on patients and healthcare systems.

C. In terms of gait assessment, we depicted wearable sensors for auto digital gait data analysis such as the 6MWT that can provide more accurate and reliable results classification of frailty individuals compared to traditional manual methods, improving the overall quality of frailty evaluations.

D. In terms of the clinical setting, the application of ML techniques to analyze and predict frailty risk has proven beneficial in identifying at-risk individuals. These methods have shown promising results in identifying relevant features and interactions, particularly when numerous variables are involved, allowing for timely interventions and more personalized care plans.

Considering the implications of a future digital health approach, we aimed to capture pertinent gait components based on 6MWT that can be utilized to remotely predict the risk of frailty using wearable sensors. With this approach, patients would able to be long-term digital monitored without having to undergo an in-person clinic visit to assess their physical frailty. However, this study is subject to several limitations that need to be addressed. Since, the 6MWT is a long-distance walking test that places higher demands on the abilities, functional status, and reserves of older adults. Our recruitment threshold of walking behavior, a minimum test time of 6 min, was achievable for the vast majority of this population with or without any walking aids. However, the physical state of all senior persons cannot be accurately represented by our community-sourced volunteer recruiting. While the physical condition of older adults in the community is generally acceptable, the degree of frailty may exhibit more pronounced manifestation within older institutions, such as nursing homes.

Throughout the research process, some patients were unable to complete the full test due to their limited capacity for sustained long-distance walking. For safety reasons, these individuals had to be excluded from the study. While we would handle missing data appropriately, this may impact the experimental results and potentially weaken the test’s effectiveness. Consequently, the restricted source of the samples supply diminishes the representativeness, universality, and generalizability of our study findings, thereby reducing their therapeutic usefulness. Although there are some intrinsic limitations to the test, the 6MWT can be potentially performed in the vast majority of geriatric population, thus key gait parameters can be served as an important tool for early frailty screening, diagnostic assessment, and early prevention in older adults (50).

Future study is warranted to validate and generalize these findings to other populations and settings, as well as to explore the potential of integrating additional data sources and advanced machine learning algorithms to further enhance the ability of ML to identify the risk of frailty. Ultimately, the insights gained from this study have the potential to significantly impact clinical practice, leading to improved identification and management of frailty in older adults, enhancing their quality of life and overall health outcomes.

## Conclusion

Overall, our findings suggest that combining CGA and wearable sensor-derived gait parameters can improve the accuracy of frailty prediction models. The use of digital measures of gait, such as the 6MWT, plays a crucial role in enhancing the model’s predictive power and should be considered by healthcare professionals when evaluating frailty risk in older patients. Due to the rapid rate at which wearable sensor-based data is being collected, high-performance data processing is becoming increasingly important. Further research is needed to determine the generalizability of these findings to other populations and settings.

## Data availability statement

The datasets presented in this article are not readily available because the datasets used and/or analyzed during the current study could be obtained from the corresponding author with reasonable requests. Requests to access the datasets should be directed to FX, xufuping1983@gzucm.edu.cn.

## Ethics statement

The studies involving human participants were reviewed and approved by Ethics Review Board of Guangdong Provincial Hospital of Chinese Medicine Ethics Committee (reference: B2017-168-01). The patients/participants provided their written informed consent to participate in this study.

## Author contributions

SF, ZY, and FX conceived and designed the study. SF, RP, and QX recruited the participants, collected the data for the manuscript and provided substantial feedback. SF, JY, ZP, and BH analyzed and interpreted the data. SF, BH, and FX wrote the first draft of the manuscript. All authors contributed to the article and approved the submitted version.

## Funding

The study was supported by the National Natural Science Foundation of China, No. 81974560; Guangzhou Science and Technology Plan Project, No. 2023A03J0736; Yan Dexin Academic Lessons learned Studio, The Second Hospital of Chinese Medicine [2014] No. 89; Scientific and technological research project of Guangdong Provincial Hospital of Chinese Medicine, No. YN2019ZWB03; Guangdong Provincial Key Laboratory of Diagnosis and Treatment of Major Neurological Diseases, No. 2020B1212060017; Guangdong Provincial Clinical Research Center for Neurological Diseases, No. 2020B1111170002; Southern China International Joint Research Center for Early Intervention and Functional Rehabilitation of Neurological Diseases, No. 2015B050501003 and 2020A0505020004; Guangdong Provincial Engineering Center for Major Neurological Disease Treatment, Guangdong Provincial Translational Medicine Innovation Platform for Diagnosis and Treatment of Major Neurological Disease, Guangzhou Clinical Research and Translational Center for Major Neurological Diseases, No. 201604020010 and Guangdong Basic and Applied Basic Research Foundation, No. 2021B1515120062. Funders played no role in this study (the design, data collection, execution, analysis, interpretation of data and writing the manuscript).

## Conflict of interest

The authors declare that the research was conducted in the absence of any commercial or financial relationships that could be construed as a potential conflict of interest.

## Publisher’s note

All claims expressed in this article are solely those of the authors and do not necessarily represent those of their affiliated organizations, or those of the publisher, the editors and the reviewers. Any product that may be evaluated in this article, or claim that may be made by its manufacturer, is not guaranteed or endorsed by the publisher.
